# Study retention and attrition in a longitudinal cohort study including patient-reported outcomes, fieldwork and biobank samples: results of the Netherlands quality of life and Biomedical cohort study (NET-QUBIC) among 739 head and neck cancer patients and 262 informal caregivers

**DOI:** 10.1186/s12874-022-01514-y

**Published:** 2022-01-22

**Authors:** Femke Jansen, Ruud H. Brakenhoff, Rob J. Baatenburg de Jong, Johannes A. Langendijk, C. René Leemans, Robert P. Takes, Chris H. J. Terhaard, Jan H. Smit, Irma M. Verdonck-de Leeuw

**Affiliations:** 1 Department of Otolaryngology-Head and Neck Surgery, Amsterdam UMC, Vrije Universiteit Amsterdam, Cancer Center Amsterdam (CCA), Amsterdam UMC, PO Box 7057, 1007 MB Amsterdam, The Netherlands; 2 Department of Otorhinolaryngology, Erasmus MC, ErasmusMC Cancer Center, University Medical Center, PO Box 2040, 3000 CA Rotterdam, The Netherlands; 3 Department of Radiation Oncology, University Medical Center Groningen, Groningen, The Netherlands; 4 Department of Otorhinolaryngology-Head and Neck Surgery, Radboud University Nijmegen Medical Center, PO Box 9101, 6500 HB Nijmegen, The Netherlands; 5 Department of Radiation Oncology, UMC Utrecht Cancer Center, Heidelberglaan 100, 3584 CX Utrecht, The Netherlands; 6 Department of Psychiatry, Neuroscience Campus Amsterdam and Amsterdam Public Health research institute, Amsterdam UMC, location VU University Medical Center, Amsterdam, The Netherlands; 7 Department of Clinical Psychology, Amsterdam Public Health, Vrije Universiteit Amsterdam, Van der Boechorststraat 1, 1081 BT Amsterdam, The Netherlands

**Keywords:** Head and neck cancer, Cohort study, Representativeness, Attrition, Retention

## Abstract

**Background:**

Longitudinal observational cohort studies in cancer patients are important to move research and clinical practice forward. Continued study participation (study retention) is of importance to maintain the statistical power of research and facilitate representativeness of study findings. This study aimed to investigate study retention and attrition (drop-out) and its associated sociodemographic and clinical factors among head and neck cancer (HNC) patients and informal caregivers included in the Netherlands Quality of Life and Biomedical Cohort Study (NET-QUBIC).

**Methods:**

NET-QUBIC is a longitudinal cohort study among 739 HNC patients and 262 informal caregivers with collection of patient-reported outcome measures (PROMs), fieldwork data (interview, objective tests and medical examination) and biobank materials. Study retention and attrition was described from baseline (before treatment) up to 2-years follow-up (after treatment). Sociodemographic and clinical characteristics associated with retention in NET-QUBIC components at baseline (PROMs, fieldwork and biobank samples) and retention in general (participation in at least one component) were investigated using Chi-square, Fisher exact or independent t-tests (*p*< 0.05).

**Results:**

Study retention at 2-years follow-up was 80% among patients alive (66% among all patients) and 70% among caregivers of patients who were alive and participating (52% among all caregivers). Attrition was most often caused by mortality, and logistic, physical, or psychological-related reasons. Tumor stage I/II, better physical performance and better (lower) comorbidity score were associated with participation in the PROMs component among patients. No factors associated with participation in the fieldwork component (patients), overall sample collection (patients and caregivers) or PROMs component (caregivers) were identified. A better performance and comorbidity score (among patients) and higher age (among caregivers) were associated with study retention at 2-years follow-up.

**Conclusions:**

Retention rates were high at two years follow-up (i.e. 80% among HNC patients alive and 70% among informal caregivers with an active patient). Nevertheless, some selection was shown in terms of tumor stage, physical performance, comorbidity and age, which might limit representativeness of NET-QUBIC data and samples. To facilitate representativeness of study findings future cohort studies might benefit from oversampling specific subgroups, such as patients with poor clinical outcomes or higher comorbidity and younger caregivers.

## Background

Longitudinal observational cohort studies in (cancer) patients are important to move research and clinical practice forward. However, study retention (continued study participation) can be compromised in such studies. Attrition (drop-out) limits the statistical power of research and, in case attrition is selective, may lead to biased results and hamper representativeness of study findings [1]. Especially in studies among cancer patients study attrition may occur due to factors such as mortality and long-term side-effects of treatment.

Previous longitudinal cohort studies provided insight into study retention and attrition among cancer patients [2–9]. Two small studies in which patients were asked to complete patient-reported outcome measures (PROMs) showed retention rates of 15% among 47 adolescent and young cancer patients (1–1.5 year follow-up) and 96% among 121 breast cancer patients (1 year follow-up) [2, 3]. In addition, Ness et al. (2016) [9] presented results of the Head and Neck 5000 study and showed that among 5356 head and neck cancer (HNC) patients 75% completed PROMs, and of 90%, 85% and 42% respectively oral rinse samples, blood samples and paraffin embedded tumor blocks were collected at baseline. At 4- and 12-months follow-up 63 and 57% of the patients completed the PROMs. In addition, Perez-Cruz et al. (2018) [6] reported that 67% of 744 advanced cancer patients showed up for a follow-up research visit at 2–5 weeks follow-up. Leuteritz et al. (2018) [5] showed that 89% of 577 young adult cancer patients completed PROMs at 1 year follow-up. A study by Ramsey et al. [7] with yearly PROM collection among 2625 colorectal cancer patients showed a retention rate of 58% of those alive (56% of all patients) at 2-years follow-up and 51% (47% of all patients) at 4 years follow-up. A study by Spiers et al. (2018) [8] among 1227 prostate cancer patients who were invited to complete PROMs at 3–6 years follow-up showed that 69% of those alive (62% of all patients) participated. Finally, Fossa et al. (2020) [4] showed that among 1436 testicular cancer patients 64% of those alive (54% of all patients) completed all three PROMs assessments over a time period of 17 years. In these studies retention rates were higher among patients who were younger [4, 7, 8], who were higher-educated [7, 8], had a higher socio-economic status [4, 7, 8], and had better clinical [4, 6, 8] and patient-reported outcomes [6, 7]. Mixed results were reported regarding gender [5–7].

Except for the study of Perez-Cruz et al. (2018) [6] and Ness et al. (2016) [9], all previous studies focused on the collection of a limited set of PROMs every 1 to 6 years. No study investigated retention and attrition in a longitudinal observational cohort study comprising different types of data, such as collection of PROMs, conduction of fieldwork assessments and collection of biobank samples, with multiple measurements per year. Also, so far, only in one study retention rates among informal caregivers of cancer patients was investigated [10]. This study of Fernix et al. (2006) [10] showed that among 206 informal caregivers of terminally ill cancer patients (all types) 85% participated in a face to face interview at 13-months follow-up.

Therefore, the aim of this study was to investigate study retention and attrition in a longitudinal cohort study with a comprehensive assessment protocol among HNC patients and their informal caregivers using data of the Netherlands Quality of Life and Biomedical Cohort Study (NET-QUBIC), and to explore sociodemographic and clinical factors associated with study retention. NET-QUBIC is a longitudinal observational cohort study among 739 HNC patients and 262 informal caregivers with collection of PROMs, fieldwork data (interview, objective tests and medical examination) and biobank materials from before start of treatment up to 2-years follow-up [11, 12]. In a previous study patients evaluated this comprehensive assessment protocol as feasible in terms of time investment and intimacy [11]. The overarching objective of NET-QUBIC is to optimize diagnosis, treatment and supportive care by advancing interdisciplinary research [11, 12]. So far, more than 20 derivate studies are ongoing or have been published that use NET-QUBIC data and samples [12–18]. Further insight in study retention and attrition will facilitate interpretation of findings derived from these studies. Also, this study contributes to designing future longitudinal cohort studies with comprehensive assessment protocols among cancer patients.

## Methods

In this study data of the NET-QUBIC observational cohort study was used [12]. Recruitment of NET-QUBIC participants took place in 7 HNC centers throughout the Netherlands. In total, 739 newly diagnosed HNC patients and 262 caregivers were recruited (March 2014 to June 2018). Inclusion criteria were previously untreated patients with newly diagnosed HNC (oral cavity, oropharynx, hypopharynx, larynx, unknown primary; all stages); age ≥ 18 years; treatment with curative intent; able to write, read, and speak Dutch. Patients were excluded in case they were diagnosed with lymphoma, skin malignancies or thyroid cancer; were unable to understand the questions or test instructions; did not provide informed consent or had severe psychiatric co-morbidities (schizophrenia, Korsakoff’s syndrome, severe dementia). A partner, family member or close friend of the patient was asked to participate as informal caregiver. The study protocol was approved by the Institutional Review Board at VUmc (2013.301(A2018.307)-NL45051.029.13) and at all local participating centers. The biobank protocol was also approved by the biobank committee of VUmc (2018.406). All patients and caregivers signed informed consent.

### NET-QUBIC assessment protocol

**Fig. 1:**
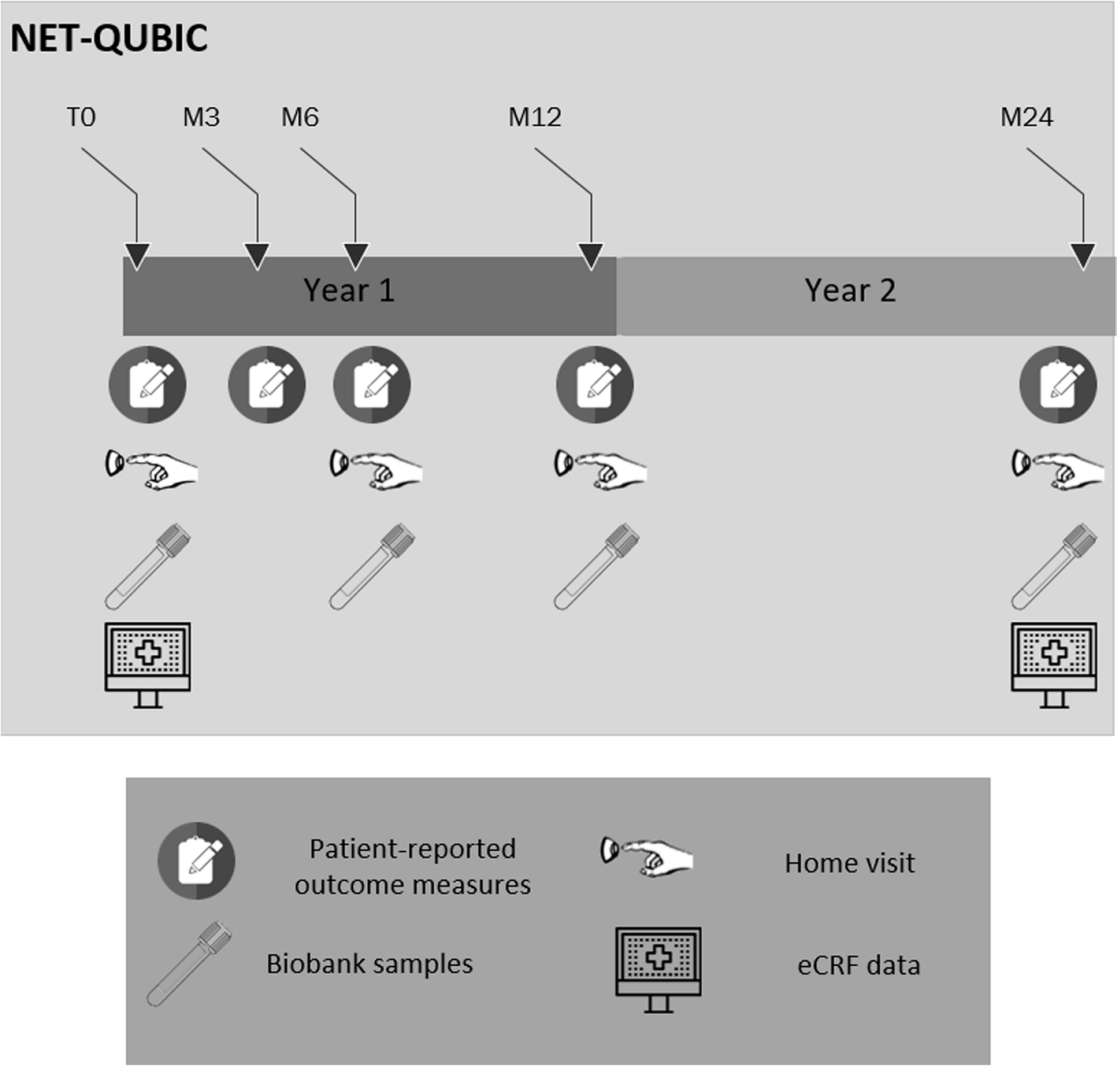
NET-QUBIC assessment protocol

In the NET-QUBIC study a wide range of measures were collected, including clinical data, PROMs, fieldwork assessments and collection of biobank samples (see Fig. 1). Clinical data was collected after the end of primary treatment, and at 2-years follow-up. PROMs were completed at baseline (approximately 2 weeks after diagnosis), and at 3 months, 6 months, 1,2, 3, 4 and 5-years after treatment by both HNC survivors and their caregivers. Fieldwork assessments including interview data, objective tests, and medical examinations were performed at baseline, 6 months, and 1–2- and 5-years follow-up among HNC survivors only. In addition, tumour biopsies were collected at baseline and biobank samples (blood, oral rinse and saliva) at baseline, 6 months, and 1-, 2- and 5-years follow-up in patients. In caregivers blood and oral rinse were collected once at baseline. These samples were collected to be used as control samples for HNC patients, as informal caregivers are likely to be comparable to HNC patients in terms of lifestyle characteristics such as alcohol consumption and smoking behaviour. For detailed insight on collected PROMs, fieldwork assessments and biobank samples we refer to previous publications [11, 12] and the NET-QUBIC website (www.kubusproject.nl). Data collection from 3- to 5-years follow-up is currently ongoing. This study, therefore, focuses on study retention and attrition up to 2 years follow-up (last 2-year follow-up assessment: August 2020).

### Study follow-up procedures

All patients and informal caregivers were asked to participate in the follow-up assessments. To limit study attrition due to loss to follow-up primary contact information was collected (name, address, phone number (home and mobile) and email address of patients and their informal caregivers. Patients and informal caregivers were asked to complete the follow-up PROMs using paper and pencil at home. Patients and informal caregivers received the PROMs by regular post and were reminded twice to complete the PROMs (also by regular post). The fieldwork assessments were scheduled by phone. In case a patient could not be reached we phoned again at a later time point. When the patient could still not be reached after several phone calls an e-mail was send to the patient (if an email address was available). To facilitate study retention the fieldwork assessments were performed at the patients’ home (with the possibility of a fieldwork assessment at the hospital when preferred by the patient). Oral rinse and blood samples were collected at time of the fieldwork assessment (preferred) or at time of clinical follow-up visit (if not willing to participate in the fieldwork assessment). Yearly newsletters were sent to patients and informal caregivers. Also, patients and their general practitioners were informed by regular post on part of the results of their fieldwork assessment and blood values (e.g. blood pressure, body mass index or haemoglobin concentration). Patients and informal caregivers were allowed to participate in a subset of NET-QUBIC components (e.g., PROMs and biobank component only) to minimize study attrition. In case patients or informal caregivers dropped-out the reason was reported.

### Statistical analyses

All analyses were performed using the IBM Statistical package for the Social Science (SPSS) version 26 (IBM Corp., Armonk, NY USA). Baseline sociodemographic and clinical characteristics of the NET-QUBIC study population were described using descriptive statistics.

**Fig. 2:**
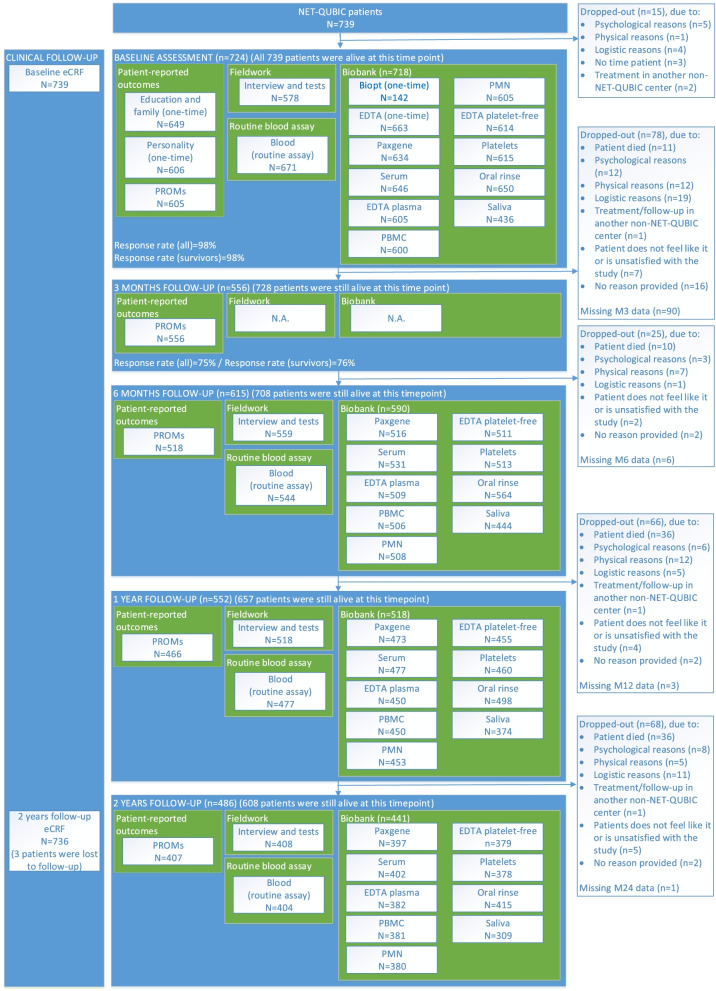
Flow diagram of NET-QUBIC patients

**Fig. 3:**
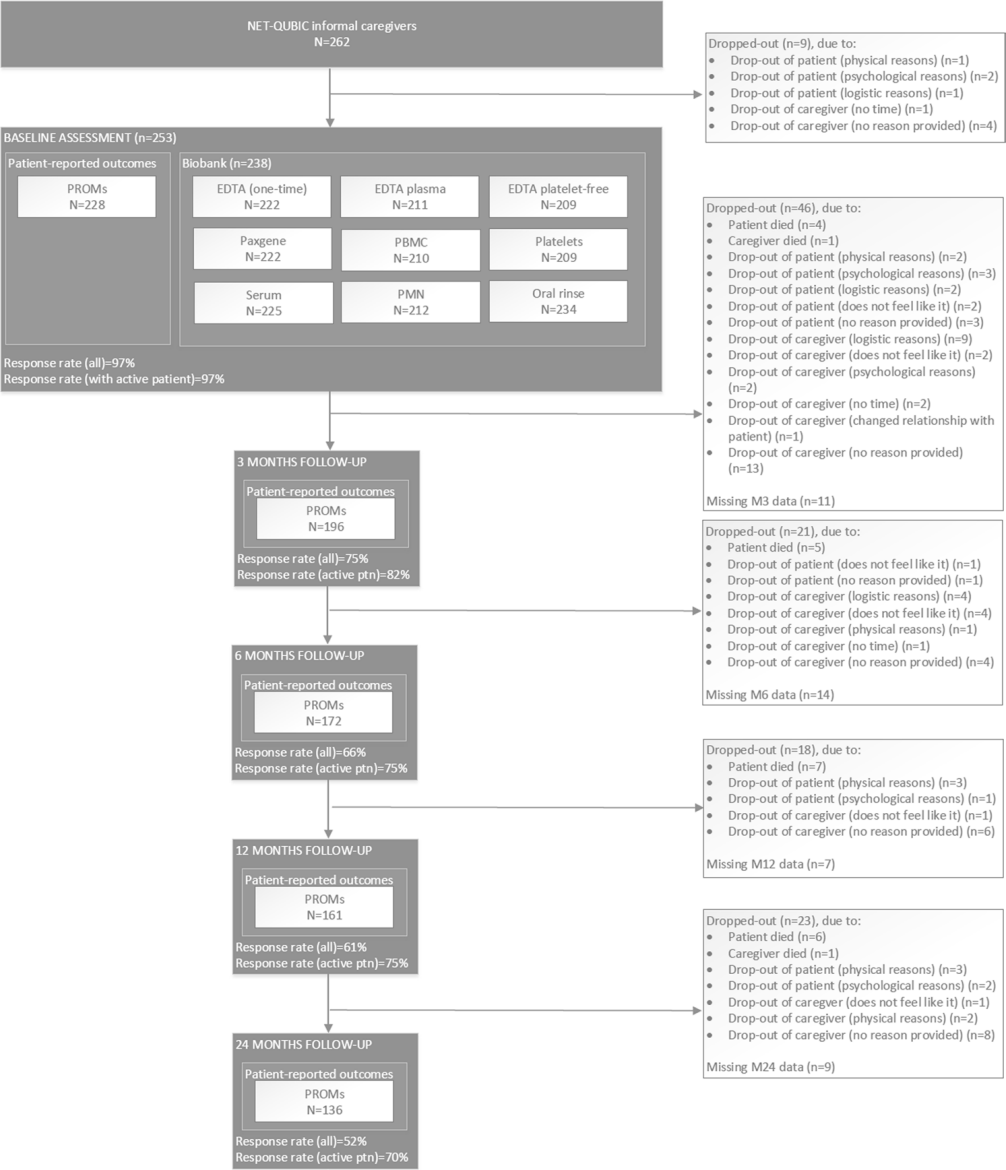
Flow diagram of NET-QUBIC informal caregivers

To describe study retention and attrition up to 2-years follow-up a detailed flow diagram is provided (Figs. 2 and 3). Patients/caregivers were coded as ‘study retention in general (yes)’ at a particular time point in case data was available on at least one NET-QUBIC component (PROMs, fieldwork, biobank). Patients/caregivers were coded as ‘study attrition (yes)’ in case no data was available on all components (PROMs, fieldwork, biobank) at this time point and all further time points. Patients/caregivers were coded as ‘drop-out: patient/caregiver died’ in case they died while still participating in the study. Other categories for drop-out were psychological reasons (e.g. distress, fatigue), physical reasons (e.g. too ill, palliative treatment), logistic reasons (e.g. patient can repeatedly not be reached), no time from the patient’s point of view, treatment or follow-up in another non-NET-QUBIC medical center or not wanting to participate anymore. The total number of patients/caregivers who died is, however, higher as some patients/caregivers dropped out due to other reasons before they died. Mortality in patients is presented separately as the number of patients alive per time point. In the group of caregivers we had no information on mortality after they dropped out. In case the patient died or dropped out, the caregiver automatically also dropped out.

Sociodemographic and clinical characteristics associated with retention in various assessment components at baseline and retention in general (participation in at least one component) at 6 months, 1- and 2-years follow-up were investigated using Chi-square tests, Fisher exact tests or independent t-tests. Factors associated with study retention at baseline were investigated for the main components (PROMs, fieldwork and biobank assessment) as well as the biobank subcomponents: tumour biopsy, blood, oral rinse and saliva. These subcomponents were studied separately as due to the study design and logistic reasons different sociodemographic and clinical factors may have influenced availability of samples. Study retention in general was investigated twice per time point: i) among all patients/caregivers and ii) among patients alive (to preclude factors associated with survival) or among caregivers of patients who were still alive and participating (to preclude factors associated with drop-out of patients). A *p*-value < 0.05 was considered statistically significant.

## Results

Previously it was shown that among 1861 patients invited for NET-QUBIC 739 patients participated (40%) [12]. In addition, 262 informal caregivers participated. Table 1 provides insight into the baseline characteristics of all patients and informal caregivers.

**Table 1:** Baseline characteristics of HNC patients and their informal caregivers

	HNC patients (***n*** = 739)	Informal caregivers (***n*** = 262)
**Mean age ± SD**	63.2 ± 9.7	58.8 ± 11.6
**Sex, n (%)**		
Men	549 (74%)	70 (27%)
Women	190 (26%)	192 (73%)
**Education level, n (%)**^**a**^		
Low	279 (43%)	82 (37%)
Middle	171 (26%)	63 (28%)
High	198 (31%)	78 (35%)
**Living situation, n (%)**^**b**^		
Living alone	163 (25%)	8 (4%)
Living with partner (with or without children)	451 (69%)	205 (91%)
Other (e.g. living with children, living with parents or assisted living	35 (5%)	12 (5%)
**Tumor location, n (%)**		
Oral Cavity	199 (27%)	
Oropharynx	262 (36%)	
Hypopharynx	52 (7%)	
Larynx	205 (28%)	
Unknown primary	21 (3%)	
**Clinical disease Stage, n (%)**		
Stage 0/I^c^	163 (22%)	
Stage II	132 (18%)	
Stage III	127 (17%)	
Stage IV	317 (43%)	
**Type of treatment, n (%)**^**d**^		
Single treatment		
Surgery	152 (21%)	
Radiotherapy	241 (33%)	
Combination treatment		
Chemoradiotherapy	215 (29%)	
Surgery and radiotherapy	106 (14%)	
Surgery and chemoradiotherapy	23 (3%)	
Other^e^	1 (0.1%)	
**WHO performance, n (%)**		
Able to carry out normal activity	507 (69%)	
Restricted in physically strenuous activity but ambulatory and able to carry out light work	191 (26%)	
Ambulatory and capable of all self-care but unable to carry out any work	40 (5%)	
Capable of only limited self-care	1 (0.1%)	
Completely disabled	0 (0%)	
**Comorbidity, n (%)**^**f**^		
None	204 (29%)	
Mild	264 (38%)	
Moderate	155 (22%)	
Severe	76 (11%)	
**HPV-status (oropharynx cancer only), n (%)**^**g**^		
Positive	99 (43%)	
Negative	130 (57%)	
**Baseline smoking status, n (%)**^**h**^		
Daily smoker	127 (22%)	38 (17%)
Not a daily smoker	445 (78%)	187 (83%)
**Baseline alcohol consumption, n (%)**^**i**^		
Excessive alcohol consumption	129 (22%)	25 (11%)
No excessive alcohol consumption	445 (78%)	200 (89%)
**Relation informal caregiver – patient, n (%)**^**j**^		
Partner		219 (84%)
Daughter/son		31 (12%)
Other (i.e. sibling, friend, ex-partner)		10 (4%)

^a^Missing in 91 patient and 39 informal caregivers. ^b^Missing in 90 patients and 37 informal caregivers. ^c^One patient had a cTNM stage of 0, however, pTNM was stage 2. ^d^One patient died before start of treatment. ^e^Radiotherapy with hyperthermic treatment. ^f^Missing in 40 patients. ^g^Missing in 33 oropharynx cancer patients. ^h^Missing in 167 patients and 37 informal caregivers. ^i^Defined as > 14 units of alcohol per week for women and > 21 units per week for men. Missing in 165 patients and 37 informal caregivers. ^j^Missing in 2 informal caregivers

### Study retention and attrition

As presented in Fig. 2, baseline clinical data was available of all 739 patients. Of the total 739 patients, 724 patients (98%) had available baseline data for PROMs, fieldwork and/or biobank samples. Fifteen patients dropped out prior to the baseline assessment due to psychological reasons (*n* = 5), logistic reasons (e.g., quick start treatment) (*n* = 4), no time from the patients point of view (*n* = 3), treatment in another non-NET-QUBIC center (*n* = 2) or physical reasons (*n* = 1). Study retention in general among all 739 patients at 3- and 6-months and 1- and 2-years follow-up was 556 (75%), 615 (83%), 552 (75%) and 486 patients (66%), respectively. Up to 2-years follow-up 131 patients died (110 patients with cancer and 21 patients with another cause). When only patients alive were taken into account study retention was 80% at 2-years follow-up (486 of 608 patients alive). At 3- and 6-months and 1- and 2-years follow-up respectively 11% (78 of 739 patients), 3% (25 of 739 patients), 9% (66 of 739 patients) and 9% (68 of 739 patients) of all patients dropped out, equaling to a total study attrition (including drop-out prior to baseline assessment) of 34% (252 of 739 patients). Most important reasons for study attrition were mortality (*n* = 93/252 (37%)), logistic reasons (*n* = 40/252 (16%)), physical reasons (*n* = 37/252 (15%)), and psychological reasons (*n* = 34/252 (13%)).

Among informal caregivers of HNC patients, of the 262 informal caregivers that signed informed consent, 253 informal caregivers completed the PROMs and/or biobank assessment at baseline (97%) (Fig. 3). Of the nine informal caregivers who did not complete the PROMs and/or biobank, reasons for non-completion were drop-out of the patient they cared for (*n* = 4) and drop-out of the informal caregiver (*n* = 5). Study retention in general among all 262 informal caregivers at 3- and 6-months and 1 and 2-years follow-up was 196 (75%), 172 (66%), 161 (61%) and 136 informal caregivers (52%) Respectively. At 3- and 6-months and 1- and 2-years follow-up, respectively 18% (46 of 262 informal caregivers), 8% (21 of 262 informal caregivers), 7% (18 of 262 informal caregivers) and 9% (23 of 262 informal caregivers) informal caregivers dropped out, equaling to a total study attrition (including drop-out prior to baseline assessment) of 45% (117 of 262 informal caregivers). Drop-out was initiated by the informal caregiver (*n* = 68/117, 58%) or followed drop-out of the patients (*n* = 49/117, 42%), of which 19% (22/117) because the patient died. When only informal caregivers were taken into account of whom the patient they cared for was still alive and participating in the study, study retention was 70% at 2-years follow-up (136 of 193 informal caregivers with an active patient).

### Factors associated with study retention

At baseline, 605 patients completed the PROMs, 578 patients the fieldwork assessment and 718 patients the biobank assessment (Fig. 2). Patients who had a less advanced tumor stage, better physical performance and lower ACE-27 comorbidity score were more likely to complete the PROMs at baseline (Table 2). There were no statistically significant differences between patients who participated in the fieldwork assessment or biobank assessment protocol (overall), and those who did not. However, some differences on subcomponents of the biobank assessment were, found. Patients with a tumor biopsy were less often diagnosed with laryngeal cancer, had a more advanced tumor stage, had a worse physical performance and had a higher comorbidity score. Blood samples were more often collected among patients who received single treatment and saliva was more often available of patients who were older and had a better physical performance score. Among informal caregivers there were no statistically significant differences between those who did and those who did not participate in the PROMs or biobank assessment at baseline (Table 3).

**Table 2:** Sociodemographic and clinical characteristics of patients that participated in the various components (PROMs, interview, biobank) and subcomponents (accelerometer, tumour biopsy, blood, oral rinse and saliva) of NET-QUBIC at baseline

	PROMs^a^ Yes *N* = 605		PROMs^a^ No *N* = 134	*P*-value	Fieldwork Yes *N* = 578		Fieldwork No *N* = 161	*P*-value	Biobank Yes *N* = 718	Biobank No *N* = 21		*P*-value
**Gender, n (%)**												
Men	450 (74%)		99 (74%)	0.90	438 (76%)		111 (69%)	0.08	536 (75%)	13 (62%)		0.19
Women	155 (26%)		35 (26%)		140 (24%)		50 (31%)		182 (25%)	8 (38%)		
**Age, mean ± SD years**	63.5 ± 9.5		62.0 ± 10.9	0.14	63.6 ± 9.3		61.9 ± 11.0	0.06	63 ± 9.7	62 ± 11.3		0.57
**Tumor location, n (%)**				0.29				0.33				0.47^c^
Oral Cavity	168 (28%)		31 (23%)		147 (25%)		52 (32%)		191 (27%)	8 (38%)		
Oropharynx	211 (35%)		51 (38%)		212 (37%)		50 (31%)		256 (36%)	6 (29%)		
Hypopharynx	38 (6%)		14 (10%)		38 (7%)		14 (9%)		52 (7%)	0 (0%)		
Larynx	169 (28%)		36 27%)		164 (28%)		41 (25%)		199 (28%)	6 (29%)		
Unknown primary	19 (3%)		2 (1%)		17 (3%)		4 (2%)		20 (3%)	1 (5%)		
**Clinical disease Stage**, n (%)				**0.011**				0.16				0.87^c^
0/I	147 (24%)		16 (12%)		133 (23%)		30 (19%)		159 (22%)	4 (19%)		
II	108 (18%)		24 (18%)		94 (16%)		38 (24%)		129 (18%)	3 (14%)		
III	97 (16%)		30 (22%)		99 (17%)		28 (17%)		122 (17%)	5 (24%)		
IV	253 (42%)		64 (48%)		252 (44%)		65 (40%)		308 (43%)	9 (43%)		
**Treatment**, n (%)^b^				0.46				0.75				0.16
Single treatment	326 (54%)		67 (50%)		306 (53%)		87 (54%)		385 (54%)	8 (38%)		
Combination treatment	279 (46%)		66 (50%)		272 (47%)		73 (46%)		332 (46%)	13 (62%)		
**WHO performance**, n (%)				**0.001**				0.51				0.72
Able to carry out normal activity	426 (70%)		81 (60%)		394 (68%)		113 (70%)		493 (69%)	14 (67%)		
Restricted in physically strenuous activity	154 (25%)		37 (28%)		154 (27%)		37 (23%)		186 (26%)	5 (24%)		
Ambulatory	25 (4%)		16 (12%)		30 (5%)		11 (5%)		39 (5%)	2 (10%)		
**Comorbidity**, n (%)				**0.003**				0.63				0.30
None	180 (31%)		24 (20%)		164 (30%)		40 (27%)		200 (29%)	4 (24%)		
Mild	223 (39%)		41 (34%)		206 (37%)		58 (39%)		257 (38%)	7 (41%)		
Moderate	117 (20%)		38 (31%)		118 (21%)		37 (25%)		153 (22%)	2 (12%)		
Severe	57 (10%)		19 (16%)		62 (11%)		14 (9%)		72 (11%)	4 (24%)		
	Tumor biopsy Yes *N* = 142	Tumor biopsy No *N* = 597	*P*-value	Blood^d^ Yes *N* = 655	Blood^d^ No *N* = 84	*P*-value	Oral rinse Yes *N* = 650	Oral rinse No *N* = 89	*P*-value	Saliva Yes *N* = 436	Saliva No *N* = 303	*P*-value
**Gender, n (%)**			0.17			0.52			0.29			0.23
Men	99 (70%)	450 (75%)		489 (75%)	60 (71%)		487 (75%)	62 (70%)		331 (76%)	218 (72%)	
Women	43 (30%)	147 (25%)		166 (25%)	24 (29%)		163 (25%)	27 (30%)		105 (24%)	85 (28%)	
**Age, mean ± SD years**	62.5 ± 10.0	63.4 ± 9.8	0.29	63.4 ± 9.6	62.3 ± 10.5	0.36	63.3 ± 9.6	62.9 ± 11.1	0.73	64.0 ± 8.9	62.2 ± 10.8	**0.022**
**Tumor location, n (%)**			**0.022**			0.58			0.20			0.26
Oral Cavity	41 (29%)	158 (26%)		173 (26%)	26 (31%)		168 (26%)	31 (35%)		105 (24%)	94 (31%)	
Oropharynx	56 (39%)	206 (34%)		232 (35%)	30 (36%)		229 (35%)	33 (37%)		166 (38%)	96 (32%)	
Hypopharynx	16 (11%)	36 (6%)		47 (7%)	5 (6%)		49 (8%)	3 (3%)		31 (7%)	21 (7%)	
Larynx	26 (18%)	179 (30%)		186 (28%)	19 (23%)		186 (29%)	19 (21%)		122 (28%)	83 (27%)	
Unknown primary	3 (2%)	18 (3%)		17 (3%)	4 (5%)		18 (3%)	3 (3%)		12 (3%)	9 (3%)	
**Clinical disease Stage**, n (%)			**0.020**			0.74			0.08			0.35
0/I	18 (13%)	145 (24%)		146 (22%)	17 (20%)		142 (22%)	21 (24%)		101 (23%)	62 (20%)	
II	28 (20%)	104 (17%)		118 (18%)	14 (17%)		121 (19%)	11 (12%)		70 (16%)	62 (20%)	
III	24 (17%)	103 (17%)		109 (17%)	18 (21%)		104 (16%)	23 (26%)		72 (17%)	55 (18%)	
IV	72 (51%)	245 (41%)		282 (43%)	35 (42%)		283 (44%)	34 (38%)		193 (44%)	124 (41%)	
**Treatment**, n (%)^b^			0.39			**0.043**			0.15			0.86
Single treatment	71 (50%)	322 (54%)		357 (55%)	36 (43%)		352 (54%)	41 (46%)		231 (53%)	162 (54%)	
Combination treatment	71 (50%)	274 (46%)		297 (45%)	48 (57%)		297 (46%)	48 (54%)		205 (47%)	140 (46%)	
**WHO performance**, n (%)			**0.028**			0.13			0.97			**0.046**
Able to carry out normal activity	86 (61%)	421 (71%)		456 (70%)	51 (61%)		445 (68%)	62 (70%)		309 (71%)	198 (65%)	
Restricted in physically strenuous activity	43 (30%)	148 (25%)		166 (25%)	25 (30%)		169 (26%)	22 (25%)		110 (25%)	81 (27%)	
Ambulatory	13 (9%)	28 (5%)		33 (5%)	8 (10%)		36 (6%)	5 (6%)		17 (4%)	24 (8%)	
**Comorbidity**, n (%)			**0.030**			0.97			0.91			0.45
None	30 (21%)	174 (31%)		180 (29%)	24 (31%)		178 (29%)	26 (32%)		129 (31%)	75 (27%)	
Mild	50 (36%)	214 (38%)		234 (38%)	30 (38%)		236 (38%)	28 (35%)		159 (38%)	105 (38%)	
Moderate	41 (29%)	114 (20%)		139 (22%)	16 (21%)		137 (22%)	18 (22%)		85 (20%)	70 (25%)	
Severe	19 (14%)	57 (10%)		68 (11%)	8 (10%)		67 (11%)	9 (11%)		46 (11%)	30 (11%)	

Patients with PROMs data, interview data or biobank samples may have missing data on components of the assessment. Groups were compared using chi square tests, unless otherwise specified. Significant *p*-values (*p* < 0.05) were printed in bold. Abbreviations: PROMs, patient-reported outcome measure, SD, standard deviation, WHO, World Health Organization

^a^The collected PROMs on education, family or personality were not taken into account, as patients were asked to complete these PROMs as part of the follow-up assessment in case data was missing

^b^One patient died before start of treatment and was therefore not included in the analysis on treatment

^c^Compared using Fisher Exact Test

^d^Including paxgene, serum, EDTA plasma, PBMC, PMN, EDTA platelet-free and/or platelets

**Table 3:** Sociodemographic and clinical characteristics of informal caregivers that participated in the various components (PROMs, biobank) of NET-QUBIC at baseline

	PROMs Yes *N* = 228	PROMs No *N* = 34	*P*-value	Biobank Yes *N* = 238	Biobank No *N* = 24	*P*-value
**Gender, n (%)**						
Men	62 (27%)	8 (24%)	0.65	61 (26%)	9 (38%)	0.21
Women	166 (73%)	26 (76%)		177 (74%)	15 (63%)	
**Age, mean ± SD years**	59.3 ± 11.3	55.7 ± 13.3	0.09	58.9 ± 11.7	57.7 ± 10.1	0.63
**Relation informal caregiver – patient**^**a**^**, n (%)**			0.14^b^			0.61^b^
Partner	194 (85%)	25 (76%)		200 (84%)	19 (83%)	
Daughter/son	26 (11%)	5 (15%)		27 (11%)	4 (17%)	
Other (i.e. sibling, friend, ex-partner)	7 (3%)	3 (9%)		10 (4%)	0 (0%)	

Informal caregivers with PROMs data or biobank samples may have missing data on specific components of the assessment. Results on the representativeness of data for a specific research question may thus differ from above results. Groups were compared using chi square tests, unless otherwise specified. Significant *p*-values (*p* < 0.05) were printed in bold

^a^Missing in two informal caregivers

^b^Compared using Fisher Exact Test

Abbreviations: *PROMs* patient-reported outcome measure, *SD* standard deviation

Sociodemographic and clinical characteristics associated with study retention in general among patients at 6-months and 1- and 2-years follow-up are presented in Table 4. Among all HNC patients, a less advanced tumor stage and a better physical performance score were associated with study retention at all time points. In addition, a better ACE-27 comorbidity score was associated with study retention at 1- and 2-years follow-up, and larynx cancer was associated with study retention at 2-years follow-up. Among HNC patients alive, better physical performance remained associated with study retention at 6-months and 1- and 2-years follow-up and ACE-27 comorbidity score with study retention at 2-years follow-up.

**Table 4:** Comparison of sociodemographic and clinical characteristics of active patients and drop-outs at 6-months, and 1- and 2-years follow-up

	6 months follow-up					1 year follow-up					2 years follow-up				
	Active patients *N* = 615	Drop-outs (all) *N* = 124	Drop-outs (excl. deceased) *N* = 93	*p*-value active vs. drop-outs (all)	*p*-value active vs. drop-outs (deceased)	Active patients *N* = 552	Drop-outs (all) *N* = 187	Drop-outs (excl. deceased) *N* = 105	*p*-value active vs. drop-outs (all)	*p*-value active vs. drop-outs (deceased)	Active patients *N* = 486	Drop-outs (all) *N* = 253	Drop-outs (excl. deceased) *N* = 122	*p*-value active vs. drop-outs (all)	*p*-value active vs. drop-outs (deceased)
**Gender**, n (%)															
Men	454 (74%)	95 (77%)	72 (77%)	0.52	0.46	409 (74%)	140 (75%)	78 (74%)	0.84	0.97	358 (74%)	191 (75%)	86 (70%)	0.59	0.48
Women	161 (26%)	29 (23%)	21 (23%)			143 (26%)	47 (25%)	27 (26%)			128 (26%)	62 (25%)	36 (30%)		
**Age**, mean ± SD years	63.4 ± 9.6	62.6 ± 10.6	61.6 ± 10.9	0.41	0.15	63.3 ± 9.6	63.1 ± 10.3	62.3 ± 10.6	0.84	0.36	63.5 ± 9.7	62.8 ± 9.8	62.1 ± 10.3	0.35	0.17
**Tumor location**, n (%)				0.82	0.94				0.46	0.49				**0.011**	0.65
Oral Cavity	165 (27%)	34 (17%)	23 (25%)			146 (26%)	53 (28%)	24 (23%)			126 (26%)	73 (29%)	35 (29%)		
Oropharynx	219 (36%)	43 (35%)	32 (34%)			197 (36%)	65 (35%)	36 (34%)			174 (36%)	88 (35%)	39 (32%)		
Hypopharynx	41 (7%)	11 (9%)	7 (8%)			34 (6%)	18 (10%)	9 (9%)			24 (5%)	28 (11%)	9 (7%)		
Larynx	171 (28%)	34 (27%)	29 (31%)			158 (29%)	47 (25%)	35 (33%)			147 (30%)	58 (23%)	37 (30%)		
Unknown primary	19 (3%)	2 (2%)	2 (2%)			17 (3%)	4 (2%)	1 (1%)			15 (3%)	6 (2%)	2 (2%)		
**Clinical disease Stage**, n (%)				**0.011**	0.11				**< 0.001**	0.12				**< 0.001**	0.50
0/I	148 (24%)	15 (12%)	14 (15%)			139 (25%)	24 (13%)	20 (19%)			126 (26%)	37 (15%)	28 (23%)		
II	113 (18%)	19 (15%)	15 (16%)			106 (19%)	26 (14%)	17 (16%)			94 (19%)	38 (15%)	22 (18%)		
III	100 (16%)	27 (22%)	22 (24%)			88 (16%)	39 (21%)	26 (25%)			76 (16%)	51 (20%)	26 (21%)		
IV	254 (41%)	63 (51%)	42 (45%)			219 (40%)	98 (52%)	42 (40%)			190 (39%)	127 (50%)	46 (38%)		
**Type of treatment,** n (%)^a^				0.28	0.65				0.06	0.86				**0.018**	0.54
Single treatment	333 (54%)	60 (49%)	48 (52%)			305 (55%)	88 (47%)	57 (54%)			274 (56%)	119 (47%)	65 (53%)		
Combination treatment	282 (46%)	63 (51%)	45 (48%)			247 (45%)	98 (53%)	48 (46%)			212 (44%)	133 (53%)	57 (47%)		
**WHO performance**, n (%)				**< 0.001**	**0.018**				**< 0.001**	**0.005**				**< 0.001**	**0.042**
Able to carry out normal activity	442 (72%)	65 (52%)	56 (60%)			400 (72%)	107 (57%)	68 (65%)			364 (75%)	143 (57%)	79 (65%)		
Restricted in physically strenuous activity	148 (24%)	43 (35%)	28 (30%)			136 (25%)	55 (29%)	27 (26%)			107 (22%)	84 (33%)	35 (29%)		
Ambulatory	25 (4%)	16 (13%)	9 (10%)			16 (3%)	25 (13%)	10 (9%)			15 (3%)	26 (10%)	8 (7%)		
**Comorbidity**, n (%)				0.06	0.32				**0.003**	0.06				**< 0.001**	**0.016**
None	179 (30%)	25 (22%)	21 (25%)			166 (32%)	38 (22%)	21 (22%)			149 (32%)	55 (23%)	25 (22%)		
Mild	226 (39%)	38 (34%)	30 (36%)			205 (39%)	59 (34%)	37 (38%)			189 (41%)	75 (32%)	41 (37%)		
Moderate	123 (21%)	32 (29%)	25 (30%)			105 (20%)	50 (29%)	30 (31%)			86 (19%)	69 (29%)	35 (31%)		
Severe	59 (10%)	17 (15%)	8 (10%)			49 (9%)	27 (16%)	9 (9%)			39 (8%)	37 (16%)	11 (10%)		

Active patients may have missing data on specific components of the assessment. Groups were compared using chi square tests. Significant *p*-values (*p* < 0.05) were printed in bold

^a^One patient died before start of treatment and was therefore not included in the analysis on treatment

Abbreviations: *SD* standard deviation, *WHO* World Health Organization

Among all informal caregivers, higher age was associated with study retention at 1- and 2-years follow-up. This association remained statistically significant among informal caregivers of patients who were still alive and participating (Table 5).

**Table 5:** Comparison of sociodemographic and clinical characteristics of active informal caregivers and drop-outs at 6-months, and 1- and 2-years follow-up

	6 months follow-up					1 year follow-up					2 years follow-up				
	Active caregivers *N* = 172	Drop-outs (all) *N* = 90	Drop-outs (excl. With inactive patients) *N* = 58	*p*-value active vs. drop-outs (all)	*p*-value active vs. drop-outs (excl. inactive)	Active caregivers *N* = 161	Drop-outs (all) *N* = 101	Drop-outs (excl. With inactive patients) *N* = 54	*p*-value active vs. drop-outs (all)	*p*-value active vs. drop-outs (excl. inactive)	Active caregivers *N* = 136	Drop-outs (all) *N* = 126	Drop-outs (excl. With inactive patients) *N* = 57	*p*-value active vs. drop-outs (all)	*p*-value active vs. drop-outs (excl. inactive)
**Gender**, n (%)				0.76	0.32				0.77	0.40				0.51	0.75
Men	47 (27%)	23 (26%)	12 (21%)			42 (26%)	28 (28%)	11 (20%)			34 (25%)	36 (29%)	13 (23%)		
Women	125 (73%)	67 (74%)	46 (79%)			119 (74%)	73 (72%)	43 (80%)			102 (75%)	90 (71%)	44 (77%)		
**Age**, mean ± SD years	59.7 ± 10.9	57.1 ± 12.7	57.2 ± 11.6	0.09	0.14	60.0 ± 10.4	56.9 ± 13.0	56.2 ± 12.6	**0.042**	**0.030**	60.9 ± 9.9	56.5 ± 12.8	55.0 ± 12.2	**0.002**	**0.002**
**Relation informal caregiver – patient**^**a**^, n (%)				0.09	0.58				0.25	0.93				0.09	0.26
Partner	151 (89%)	68 (77%)	48 (83%)			140 (87%)	79 (80%)	46 (85%)			121 (89%)	98 (79%)	46 (81%)		
Daughter/son	16 (9%)	15 (17%)	7 (12%)			15 (9%)	16 (16%)	6 (11%)			11 (8%)	20 (16%)	7 (12%)		
Other (i.e. sibling, ex-partner)friend,	5 (3%)	5 (6%)	3 (5%)			6 (4%)	4 (4%)	2 (4%)			4 (3%)	6 (5%)	4 (7%)		

Informal caregivers with PROMs data or biobank samples may have missing data on specific components of the assessment. Results on the representativeness of data for a specific research question may thus differ from above results. Groups were compared using chi square tests, unless otherwise specified. Significant *p*-values (*p* < 0.05) were printed in bold

^a^Missing in two informal caregivers

Abbreviations: *PROMs* patient-reported outcome measure, *SD* standard deviation

## Discussion

The aim of this study was to investigate study retention and attrition, and sociodemographic and clinical factors associated with study retention up to two years follow-up in a longitudinal cohort study among HNC patients and their informal caregivers. At 2-years follow-up, study retention was 80% among patients alive at that time-point (66% among all included HNC patients) and 70% among informal caregivers with a patient still actively participating in the study (52% among all included informal caregivers). Reasons for study attrition among HNC patients were mostly mortality-related, followed by logistic, physical, or psychological factors. Both sociodemographic and clinical outcomes influenced participation in the different NET-QUBIC (sub) components (PROMs, tumour biopsy, blood, and saliva) at baseline. Study retention in general (participation in at least one NET-QUBIC component) at 2-years follow-up was associated with a better physical performance and lower comorbidity score (patients still alive) and younger age (caregivers).

The NET-QUBIC retention rate of 66–80% among HNC patients is relatively high compared to the retention rate over time of 56% (among all patients included at baseline) to 58% (among all patients included at baseline and believed to be alive) at 2-years follow-up found in a previous observational cohort study among colorectal cancer patients (recalculated) [7]. It is also high in comparison to another large observational cohort study (i.e. Head and Neck 5000) which showed that 57% of HNC patients completed PROMs at 1-years follow-up [9]. A possible explanation might be that, by study design, we facilitated study retention by performing the fieldwork assessment at the patients’ home (and the possibility of a fieldwork assessment at the hospital when preferred by the patient), by sending reminders by post in collecting PROMs data, and by sending yearly newsletters to patients and informal caregivers. A previous meta-analysis on the effectiveness of retention strategies in both clinical and non-clinical observational cohort studies also showed that offering site and home visits might improve study retention. However, no effects of newsletters and reminders were found [1]. The high retention rate in the NET-QUBIC study might also result from the design of the NET-QUBIC study, in which patients were allowed to participate in a subset of NET-QUBIC components (e.g., fieldwork and biobank component only) to minimize study attrition. When we focus on the collection of PROMs, 67% of patients alive participated at 2-years follow-up, whereas 67% of patients alive participated in the fieldwork component and 73% in the biobank component. These high percentages per NET-QUBIC component support the previous findings of van Nieuwenhuizen et al. [11], who showed that a comprehensive assessment protocol as used in the NET-QUBIC study is evaluated as feasible by patients in terms of time investment and intimacy. The NET-QUBIC retention rate of 61 and 52% (all informal caregivers) to 75 and 70% (all informal caregivers with a patient still actively participating in the study) at respectively 1 and 2-years follow-up is lower than the retention rate of 85% at 13-months follow-up reported in a study in which face to face interviews were conducted among informal caregivers of terminally ill patients [10]. An explanation for this difference might be that informal caregivers are more likely to participate in face to face interviews in comparison to completing PROMs (in NET-QUBIC informal caregivers only filled in PROMs at the follow-up time points), but more research is needed on this hypothesis.

The most important reason for study attrition among HNC patients in the NET-QUBIC study was mortality, followed by logistic, physical or psychological reasons, which are also common reasons for study attrition in clinical trials among cancer patients [19, 20]. In contrast to previous findings [4, 7, 8], age was not associated with study retention in general. In line with previous studies [4, 6, 8], however, better clinical outcomes such as a better physical performance and favourable comorbidity score were found to be associated with study retention. Together with previous results of Verdonck-de Leeuw et al. (2019) [12] who showed that HNC patients who participated in the NET-QUBIC study were younger, more often male, diagnosed with oropharyngeal cancer, and treated with radiotherapy and chemotherapy (compared to all HNC patients diagnosed), these findings indicate that NET-QUBIC data and samples might be slightly selective. Also, data and samples of informal caregivers of HNC patients are not entirely representative, as older caregivers were more likely to continue study participation. Future NET-QUBIC researchers should be aware of these differences in participation and retention when writing the data analysis plan of their NET-QUBIC study and reflect on the representativeness of their findings in the discussion section. In addition, future cohort studies might benefit from oversampling specific subgroups such as patients with poor clinical outcomes or younger caregivers.

Besides differences in study retention over time, some minor differences were found between patients who did and who did not participate in the different NET-QUBIC (sub) components at baseline. Patients with worse clinical outcomes (e.g., worse physical performance) were less likely to complete PROMs and to participate in saliva collection. These (sub) components needed to be performed by the patient without the researcher/fieldworker present, which might be more difficult for patients with a poorer physical performance (e.g., due to inability to open saliva tubes or to post the PROMs/saliva tubes). Tumor biopsies, however, were more often collected among patients with worse tumor-specific and clinical outcomes. This might be explained by the NET-QUBIC study design, in which additional tumor biopsies were only collected in case tumor biopsies were already collected for diagnostics purposes and sufficient tumor tissue was available. Finally, older age was associated with higher participation in the collection of saliva samples. Only patients who participated in the fieldwork component were asked to collect saliva samples the following day. The higher age among participants of the saliva component might, consequently, directly result from the higher age among participants of the fieldwork assessment (although non-significant, *p*-value of 0.06). Our hypothesis is that working-aged patients are less likely to participate in the fieldwork/saliva (sub) component due to work obligations. These differences among participants and non-participants might limit representativeness of NET-QUBIC data and samples. However, it might also improve representativeness of NET-QUBIC data. For example, the older age among patients who participated in the fieldwork or saliva (sub) component may counteract the lower recruitment rate among older HNC patients found by Verdonck-de Leeuw et al. [12].

To improve retention rates in future longitudinal cohort studies in cancer patients and their informal caregiver, we, suggest to personalize the method of participation and offer both online and paper and pencil options to complete the PROMs. In the NET-QUBIC study we asked all patients and informal caregivers to complete the PROMs using paper and pencil, as it was estimated that about 30% quite some HNC patients have no internet or limited computer skills has no access to the internet or limited computer skills [21, 22]. Some, potentially younger, HNC patients and informal caregivers may, however, be more likely to complete PROMs on a computer or smartphone. In addition, although we have no data to support this assumption, we assume that the involvement of a dedicated research nurse or research coordinator for a long period of time is of great importance for study retention.

A strength of this study is that it is the first study that investigated study retention and attrition in a longitudinal cohort study among cancer patients with PROMs, fieldwork and biobank assessments. A limitation of this study is, however, that, due to the design of the study, only sociodemographic and clinical characteristics could be compared among groups of patients and caregivers (and not PROMs). Also, sociodemographic and clinical characteristics of NET-QUBIC participants were only compared for the main NET-QUBIC component (PROMs, fieldwork, biobank) and a selection of NET-QUBIC subcomponents (tumor biopsy, blood, oral rinse and saliva). Due to patient or caregiver-related factors specific PROMs or subcomponents of the fieldwork assessment may, however, be missing and influence representativeness of data (e.g., specific PROM questions on sexuality may be left open by certain groups of patients or informal caregivers).

## Conclusions

Retention rates of the NET-QUBIC study are high. Nevertheless selection may have occurred during the study, which might slightly limit representativeness of NET-QUBIC data and samples. To facilitate representativeness of study findings future cohort studies might benefit from oversampling specific subgroups such as patients with poor clinical outcomes or higher comorbidity and younger caregivers.

## Data Availability

The datasets generated and analyzed during the current study are not publicly available as the collection and integration of large amounts of personal, biological, genetic and diagnostic information precludes open access to the NET-QUBIC research data. Data are available from the corresponding author on reasonable request. On the NET-QUBIC website (www.kubusproject.nl) is described how NET-QUBIC data are made available for the research community.

## Abbreviations

NET-QUBIC: Netherlands Quality of Life and Biomedical Cohort Study
HNC: Head and neck cancer
PROMs: Patient-reported outcome measures
ACE-27: Adult Comorbidity Evaluation 27
WHO: World Health Organization
HPV: Human papillomavirus
